# Does Cardiopulmonary Testing Help Predict Long-Term Survival After Esophagectomy?

**DOI:** 10.1245/s10434-021-10136-5

**Published:** 2021-05-26

**Authors:** Jakub Chmelo, Rachel A. Khaw, Rhona C. F. Sinclair, Maziar Navidi, Alexander W. Phillips

**Affiliations:** 1 Northern Oesophagogastric Unit, Royal Victoria Infirmary, Newcastle Upon Tyne, UK; 2 Department of Anaesthesia and Critical Care Medicine, Royal Victoria Infirmary, Newcastle Upon Tyne, UK; 3 School of Medical Education, Newcastle University, Newcastle Upon Tyne, UK

## Abstract

**Background:**

Esophagectomy is associated with a high rate of morbidity and mortality. Preoperative cardiopulmonary fitness has been correlated with outcomes of major surgery. Variables derived from cardiopulmonary exercise testing (CPET) have been associated with postoperative outcomes. It is unclear whether preoperative cardiorespiratory fitness of patients undergoing esophagectomy is associated with long-term survival. This study aimed to evaluate whether any of the CPET variables routinely derived from patients with esophageal cancer may aid in predicting long-term survival after esophagectomy.

**Methods:**

Patients undergoing CPET followed by trans-thoracic esophagectomy for esophageal cancer with curative intent between January 2013 and January 2017 from single high-volume center were retrospectively analyzed. The relationship between predictive co-variables, including CPET variables and survival, was studied with a Cox proportional hazard model. Receiver operation curve (ROC) analysis was performed to find cutoff values for CPET variables predictive of 3-year survival.

**Results:**

The study analyzed 313 patients. The ventilatory equivalent for carbon dioxide (VE/VCO_2_) at the anerobic threshold was the only CPET variable independently predictive of long-term survival in the multivariable analysis (hazard ratio [HR], 1.049; 95% confidence interval [CI], 1.011–1.088; *p* = 0.011). Pathologic stages 3 and 4 disease was the other co-variable found to be independently predictive of survival. An ROC analysis of the VE/VCO_2_ failed to demonstrate a predictive cutoff value of 3-year survival (area under the curve, 0.564; 95% CI, 0.499–0.629; *p* = 0.056).

**Conclusions:**

A high VE/VCO_2_ before esophagectomy for malignant disease is an independent predictor of long-term survival and may be an important variable for clinicians to consider when counseling patients.

Esophagectomy, the cornerstone for curative treatment of patients with esophageal cancer,^1^ is associated with high rates of morbidity and mortality (2.1%).^2^ Preoperative cardiopulmonary fitness has been correlated with outcomes in various types of surgery. These have indicated that less fit patients have a higher incidence of postoperative morbidity and mortality.^3^,^4^

Many centers use cardiopulmonary exercise testing as a method of individualized risk assessment before major surgery. Severe complications in the postoperative period after esophagectomy may reduce long-term survival.^5^ Variables derived from cardiopulmonary exercise testing (CPET) such as the anaerobic threshold and peak oxygen uptake (VO_2peak_) have been associated with an increased chance of complications developing after esophagogastric cancer surgery, although study results are conflicting.^6^–^17^

It is unclear whether the preoperative cardiorespiratory fitness of patients undergoing esophagectomy is associated with intermediate or long-term survival. This study aimed to evaluate whether any of the CPET variables routinely derived in patients with esophageal cancer may aid in predicting long-term survival after esophagectomy.

## Methods

### Patient Population

A contemporaneously maintained database of all patients with adenocarcinoma or squamous cell carcinoma of the esophagus or gastroesophageal junction was reviewed. The study investigated patients undergoing esophagectomy for esophageal cancer with curative intent between January 2013 and January 2017 at the Northern Oesophagogastric Unit, Newcastle upon Tyne. Previous studies have evaluated the association of CPET variables and perioperative morbidity, but the main aim of this study was to investigate the association of these variables with long-term survival. To this end, patients who died within 30 days after surgery were excluded from survival calculations.

### Staging and Treatment

Initial cancer staging comprised endoscopy with biopsy, endoscopic ultrasonography, and a thoraco-abdominal computed tomography scan and positron emission tomography. Operative fitness was assessed using cardiopulmonary exercise testing.^18^,^19^ Patients with locally advanced tumors (≥T3, any node-positive disease) were considered for perioperative chemotherapy with ECX^20^ or neoadjuvant chemoradiation therapy using the CROSS protocol.^21^ Current Union for International Cancer Control TNM 8 was used to stage all the patients in this study. Comorbidities were scored using the Charlson Comorbidity Index.^22^,^23^

The majority of the resections were performed using a standardized two-phase transthoracic approach (Ivor Lewis) with a radical en bloc abdominal and mediastinal lymphadenectomy as previously described.^24^ A small number of patients had a three-stage procedure with neck anastomosis, and a single patient was treated with a left thoraco-abdominal approach (Table 1). For the majority of the patients, this was performed as an open procedure, but a small number of patients had a thoracoscopic chest phase with an equivalent lymphadenectomy.

**Table 1:** Demographics of the patients^a^

Median age at operation: years (IQR)		66 (60–71)
Gender, male		236 (75.4)
ASA	1	22 (7.0)
	2	183 (58.5)
	3	108 (34.5)
Median VE/VCO_2_ (IQR)		29 (27–32)
Median AT: ml min^–1^ kg^–1^ (IQR)		14.1 (12.2–17.3)
Median VO_2peak_: ml min^–1^ kg^–1^ (IQR)		19.2 (16.2–22.7)
Median CCI: *n* (IQR)		4 (4–5)
Histology, AC		232 (74.1)
Neoadjuvant treatment, yes		232 (74.1)
Operation type	Ivor Lewis	292 (93.3)
	McKeown	20 (6.4)
	Left thoraco-abdominal	1 (0.3)
Pathologic stage	0	27 (8.6)
	1	61 (19.5)
	2	74 (23.6)
	3	113 (36.1)
	4	38 (12.1)
Tumor regression grade	1	27 (8.6)
	2	18 (5.8)
	3	62 (19.8)
	4	100 (31.9)
	5	21 (6.7)
	Unknown	85 (27.2)
Longitudinal resection margin, R1		8 (2.6)

*IQR* interquartile range, *ASA* American Society of Anesthesiologists physical status classification system, *VE/VCO*_*2*_ ventilatory equivalents of carbon dioxide, *AT* anaerobic threshold, *VO*_*2peak*_ peak oxygen uptake, *CCI* Charlson Comorbidity Index, *AC* adenocarcinoma

^a^Values in parenthesis are percentages unless indicated otherwise.

All the patients were managed in the perioperative period with an enhanced recovery after surgery program (ERAS). After discharge from the hospital, the patients were routinely followed up in the outpatient clinic. Follow-up reviews were initially performed in 3- to 6-month intervals during the first 2 years and annually thereafter unless clinical factors determined a more frequent follow-up evaluation. Patient mortality was recorded from the hospital electronical system or the general practitioner record on 14 July 2020, providing a minimum follow-up interval of 42 months.

### Cardiopulmonary Exercise Testing

At the Northern Oesophagogastric Unit (NOGU), CPET has been used to assess patients potentially undergoing esophagectomy since January 2013. This test forms part of the initial assessment, and the results are used to assist in the tumor board treatment decisions. All the patients in this study had completed at least 3 years of follow-up evaluation since surgery. The patients’ CPET data, demographics, and information relating to their disease, operation, postoperative period, and survival were analyzed.

In this study, CPET was performed in accordance with the American Thoracic Society/American College of Chest Physicians guidelines for this testing.^19^ These guidelines also were used to exclude patients who had a contraindication to undergoing CPET. Each test was performed according to a local protocol based on that described by Older et al.^3^ The patients performed a continuous pedaling ramped test on a cycle ergometer (Ergoselect 200, Ergoline GmbH, Bitz, Germany) until they were physically exhausted, had reached VO_2max_, or had to discontinue the test due to clinical indications.

A standard 12-lead electrocardiogram (ECG) with ST segment analysis together with pulse oximetry (Welch Allyn, Skaneateles Falls, New York, NY, USA) was used throughout the test. Metabolic gas analysis was performed via the metabolic cart (Ultima Series; MGC Diagnostics, Saint Paul, MN, USA). The CPET data were analyzed using the Breeze SuiteTM software (Ultima Series; MGC Diagnostics). The tests were reported by a consultant anesthetist trained in reporting CPET.

The VO_2peak_ was defined as the highest oxygen consumption recorded at volitional exhaustion during the last 30 s of the exercise. The anaerobic threshold was defined by the V-slope method representing the amount of VO_2_ during a ramped test above which aerobic energy production is supplemented by anaerobic mechanisms.^25^ The ventilatory equivalents of carbon dioxide (VE/VCO_2_) were determined using linear regression analysis of VE and VCO_2_ at the anaerobic threshold level.^26^ If the anaerobic threshold was not reached, VE/VCO_2_ was recorded as the lowest value observed.

### Statistical Analysis

Statistical analysis was performed using IBM SPSS Statistics version 26 (IBM, Armonk, USA). A multivariable Cox regression model was used to establish the relationship between predictive variables and survival. Variables with a *p* value lower than 0.1 in the univariable analysis were inserted into the model. Survival was estimated using Kaplan–Meier survival curves and compared using the log-rank test. Receiver operation curve (ROC) analysis was conducted for CPET variables that were independently predictive of survival in the Cox regression model. Survival status at 3 years was used as a dependent binary variable in the ROC analysis. A *p* value lower than 0.05 was deemed statistically significant.

## Results

During the study period, 318 patients underwent esophagectomy with curative intent. Five patients were excluded due to death within the first 30 days after surgery. The remaining 313 patients were analyzed in this study. Of these 313 patients, 236 (75.4%) were male, and the median age was 66 years (range, 42–84 years). The majority of the patients received neoadjuvant treatment (74.1%). The characteristics for the entire cohort are shown in Table 1.

The median survival time was 57 months. The factors found to be predictive of survival in the univariable analysis are listed in the Table 2.

**Table 2:** Cox univariable regression analysis of the factors influencing survival^a^

	HR	95% CI	*p* Value
Male gender	**1.424**	**0.961–2.111**	**0.078**
CCI	**1.192**	**1.039–1.367**	**0.012**
Neoadjuvant treatment, yes	1.278	0.878–1.860	0.201
Age at operation	1.015	0.995–1.035	0.150
ASA 1	Reference		
2	1.247	0.649–2.398	0.508
3	1.424	0.728–2.788	0.302
VE/VCO_2_	**1.054**	**1.017–1.093**	**0.004**
AT	0.991	0.955–1.029	0.635
VO_2peak_	0.987	0.957–1.017	0.391
Histology, AC	**1.572**	**1.061–2.329**	**0.024**
Longitudinal resection margin, R1	**2.623**	**1.154–5.959**	**0.021**
Pathologic stage 0	Reference		
1	0.577	0.205–1.620	0.494
2	1.277	0.518–3.150	0.467
**3**	**5.351**	**2.331–12.285**	**<0.001**
**4**	**10.518**	**4.399–25.150**	**<0.001**
Tumor regression grade 1	Reference		
2	1.152	0.351–3.774	0.816
3	1.490	0.601–3.692	0.389
**4**	**4.403**	**1.911–10.143**	**<0.001**
**5**	**5.310**	**2.091–13.489**	**<0.001**
**Unknown**	**2.280**	**0.964–5.394**	**0.061**
Clavien-Dindo complications grade None	Reference		
1–2	0.924	0.647–1.319	0.662
3–4	1.378	0.853–2.225	0.190

*HR* hazard ratio, *CI* confidence interval, *CCI* Charlson Comorbidity Index, *ASA* American Society of Anesthesiologists physical status classification system, *VE/VCO*_*2*_ ventilatory equivalents of carbon dioxide, *AT* anaerobic threshold, *VO*_*2peak*_ peak oxygen uptake, *AC* adenocarcinoma

^a^Bold indicates co-variables entered into multivariable model.

In the univariable analysis, the VE/VCO_2_ at the anaerobic threshold was the only CPET variable significantly predictive of survival (hazard ratio [HR], 1.054; 95% confidence interval [CI], 1.017–1.093; *p* = 0.004). The anaerobic threshold (HR, 0.991; 95% CI, 0.955–1.029; *p* = 0.635) and VO_2peak_ (HR, 0.987; 95% CI, 0.957–1.017; *p* = 0.391) were not found to be associated with survival.

The multivariable analysis showed that VE/VCO_2_ (HR, 1.049; 95% CI, 1.011–1.088; *p* = 0.011) is a significant independent predictor of overall survival. In the multivariable model, pathologic stages 3 and 4 disease was the other co-variable found to be independently predictive of survival (Table 3).

**Table 3:** Cox multivariable regression analysis of the factors influencing survival^a^

	HR	95% CI	*p* Value
Male gender	1.511	0.985–2.319	0.059
CCI	1.138	0.985–1.313	0.078
VE/VCO_2_	**1.049**	**1.011–1.088**	**0.011**
Histology, AC	1.444	0.939–2.220	0.094
Longitudinal resection margin, R1	0.725	0.307–1.716	0.465
Pathologic stage 0	Reference		
1	0.594	0.079–4.490	0.614
2	1.842	0.266–12.778	0.536
**3**	**7.545**	**1.070–53.188**	**0.043**
**4**	**15.172**	**2.100–109.600**	**0.007**
Tumor regression grade 1	Reference		
2	0.342	0.041–2.876	0.323
3	0.352	0.048–2.595	0.306
4	0.632	0.088–4.528	0.648
5	0.639	0.084–4.861	0.665
Unknown	0.831	0.120–5.755	0.851

*HR* hazard ratio, *CI* confidence interval, *CCI* Charlson Comorbidity Index, *VE/VCO*_*2*_ ventilatory equivalents of carbon dioxide, *AC* adenocarcinoma

^a^Bold indicates significant findings.

**Fig. 1:**
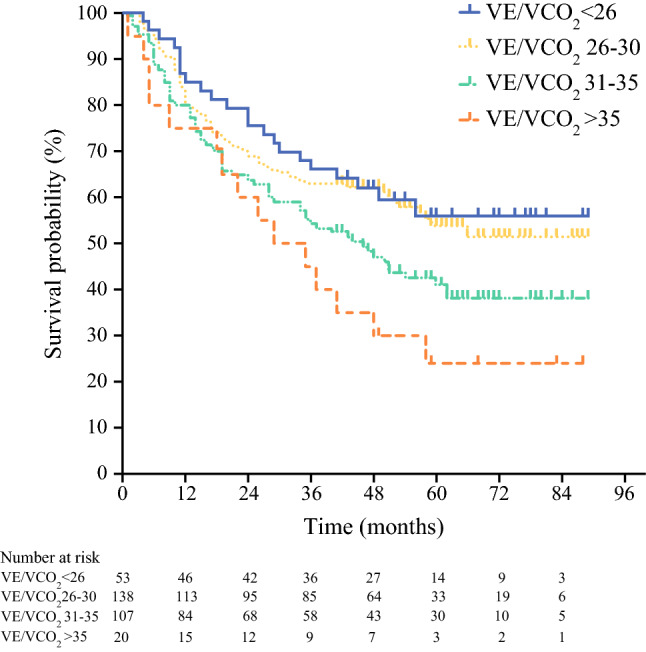
Kaplan–Meier curves showing survival according to VE/VCO_2_

The survival curves indicated shorter survival with higher VE/VCO_2_ when the patients were stratified into groups according to their VE/VCO_2_ value (Fig. 1). The median survival time was not reached for the group with a VE/VCO_2_ lower than 26 or for the group with a VE/VCO_2_ of 26 to 30. It was 46 months for the group with a VE/VCO_2_ of 31 to 35 and 29 months for the group with a VE/VCO_2_ higher than 35. The difference between the groups was found to be statistically significant (*p* = 0.015).

The ROC analysis of VE/VCO_2_ failed to demonstrate a predictive cutoff value that would divide patients into groups with a low or high chance of a 3-year survival period (area under the curve [AUC], 0.564; 95% CI, 0.499–0.629; *p* = 0.056).

## Discussion

The results from this study indicate that ventilatory inefficiency (high VE/VCO_2_) is associated with long-term survival for patients undergoing esophagectomy for malignant disease. The overall 30-day mortality rate was 1.9%, and the 90-day mortality rate was 3.1%. When patients are stratified according to their VE/VCO_2_, a clear association of higher (poorer) VE/VCO_2_ is observed, with worse long-term survival.

Previously, VE/VCO_2_ was linked with pulmonary morbidity after lung resection^27^ and abdominal aortic repair surgery.^28^ However, the data for patients undergoing esophagectomy is sparse despite the high morbidity and physiologic demand of this procedure on a patient’s reserve. These results are similar to the findings of Wilson et al.,^29^ who examined 1375 patients undergoing colorectal cancer surgery and demonstrated that VE/VCO_2_ was independently associated with 90-day mortality and long-term survival. Apart from the evidence of spread during the surgery (indicating metastatic disease), this study of colorectal patients did not consider other oncologic factors that might influence long-term survival. Importantly, in the current study, after adjustment for several oncologic factors (neoadjuvant therapy, pathologic stage of the disease, resection margin involvement, and tumor regression grade), VE/VCO_2_ remained an independent predictor of survival. In addition, the comorbidities of the patients were considered, and when the Charlson Comorbidity Index was used to adjust for these comorbidities, they were not found to be associated with overall survival. The reasons for this may be that a minimum yet sufficient level of fitness must exist before a patient is considered for esophagectomy, with less fit patients commonly excluded from surgery.

The results of this study confirm that a high VE/VCO_2_ should remain a trigger for further detailed investigations of an individual’s health before esophagectomy. An elevated VE/VCO_2_ should prompt consideration of asymptomatic heart failure, chronic obstructive pulmonary disease (COPD), or interstitial pulmonary disease. In the current cohort, only one patient had presented with a clinical diagnosis of heart failure, but by CPET criteria, other patients had CPET-defined “heart failure.” Patients with a diagnosis of heart failure usually are not deemed sufficiently fit to undergo esophagectomy without being optimized. Therefore, VE/VCO_2_ can be used to stratify patients into low- and high-risk categories and may potentially identify a group that can be optimized before surgery.

Patients with poor cardiopulmonary function have less physiologic reserve and are less able to cope with postoperative complications. The literature contains conflicting results as to whether complications after esophagogastric surgery lead to poorer long-term survival.^30^–^32^ In the current study, severe complications, defined as Clavien-Dindo grade 3 or higher, within 30 days after surgery were not related to survival in the univariable analysis.

Interestingly, these data did not demonstrate any association of survival with other commonly reported CPET variables such as the anaerobic threshold (*p* = 0.635) or VO_2peak_ (*p* = 0.391). The findings are conflicting concerning the use of CPET variables for prediction of postoperative mortality after esophagogastric surgery. Both Whibley et al.^12^ and Benington et al.^15^ demonstrated an association between the anaerobic threshold, VO_2peak_, and mortality after esophagogastric resections. Jack et al.^33^ found this association only for the pre-neoadjuvant chemotherapy anaerobic threshold. These studies had smaller samples than the current analysis, concentrated mainly on early mortality, demonstrated association with univariable analyses, or used dichotomized rather than continuous variables, which can lead to misleading results.^34^ In contrast, Patel et al.^16^ did not demonstrate any association between these variables and mortality. The failure of the anaerobic threshold and the VO_2peak_ to reach significance in the current study might have been due to the higher baseline values of these parameters. The median anaerobic threshold of 14.1 ml/min^-1^/kg^-1^ and the VO_2peak_ of 19.2 ml/min^-1^/kg^-1^ rendered this cohort more fit than the cohorts in some other studies. This study also aimed to investigate long-term survival. The anaerobic threshold and the VO_2peak_ are commonly associated with poor perioperative outcomes for patients after major surgery.^4^ The pathologic stage of the disease is one of the strongest predictors of survival in esophageal cancer. The findings regarding VE/VCO_2_ are relevant for patient counseling because they identify another factor that could help to inform clinicians and patients on long-term prognosis. Although the ROC analysis did not determine a predictive cutoff for identifying higher-risk patients, a clear trend emerged when the patients were stratified according to the VE/VCO_2_. For the patients with a VE/VCO_2_ higher than 35, the median survival time was 29 months compared with 46 months for those with a VE/VCO_2_ of 31 to 35. Therefore, different and less aggressive treatment options that maintain higher post-treatment quality of life might be sought for patients with ventilatory inefficiency.

This study had a number of limitations. It was a retrospective study with its inherent limitations. However, the data were collected contemporaneously. Although this was the largest study to assess CPET variables in esophageal cancer surgery, it was not possible to identify cutoff values of VE/VCO_2_ suggesting which patients are more likely to survive for 3 years. Furthermore, no data exist to show the patients who might have been excluded based on their CPET results. It is expected that many patients with ventilatory inefficiency never progressed to surgery. Although neoadjuvant treatment leads to longer overall survival,^20^, ^21^ some patients who have become deconditioned during chemotherapy^35^ might actually survive for a shorter period as a result. The CPET data analyzed in this study were measured before any oncologic treatment. Thus, these values did not account for the impact of neoadjuvant chemotherapy treatment on cardiopulmonary fitness. However, previous findings have demonstrated that VE/VCO_2_ remains unchanged during the neoadjuvant part of the treatment.^35^

The study deliberately excluded patients who died within 30 days after surgery because the focus was on long-term survival. However, the study chose to include the patients who died within 90 days. There were two main reasons for this. First, it was thought that these patients may have died due to postoperative complications they struggled to overcome because of a worse baseline fitness instead of a more sudden acute complication. Second, those with a short survival not curtailed by complications conceivably could have died within this time frame.

Controversy remains within the available published literature surrounding CPET variables and outcome prediction. More studies with larger samples are needed to investigate this for further improvement in the clinical utility of CPET before esophageal surgery.

However, the large cohort of patients that underwent esophageal surgery in the current study demonstrated that VE/VCO_2_ does have a role in prognostication.
